# Characterization, Hypoglycemic Activity, and Antioxidant Activity of Methanol Extracts From *Amomum tsao-ko*: *in vitro* and *in vivo* Studies

**DOI:** 10.3389/fnut.2022.869749

**Published:** 2022-07-12

**Authors:** Libin Xie, Dan Yu, Yanan Li, Huidong Ju, Jia Chen, Lianxia Hu, Longquan Yu

**Affiliations:** ^1^Shijiazhuang Food Engineering Technology Research Center, School of Chemical Engineering, Shijiazhuang University, Shijiazhuang, China; ^2^Department of Nutrition, Third Hospital of Hebei Medical University, Shijiazhuang, China

**Keywords:** *Amomum tsao-ko*, methanol extracts, hypoglycemic effect, antioxidant effect, mice

## Abstract

The dried fruit of *Amomum tsao-ko* is well-known as a spice as well as a Chinese traditional herb. This study aimed to identify the bioactive constituents in the powder of methanol extract from *Amomum tsao-ko* (PMEAT) and to evaluate the hypoglycemic and antioxidant effects of PMEAT, *in vitro* and *in vivo*. We identified 36 phytochemicals in PMEAT by employing HPLC-MS/MS. PMEAT solution was found to have potent α-glucosidase-inhibiting activity (IC_50_, 0.145 mg/mL) *in vitro*, twice as strong as that of acarbose (IC_50_, 0.273 mg/mL). To investigate the hypoglycemic activity of PMEAT *in vivo*, we studied the impact of low-dose PMEAT (the addition of 100 mg/kg PMEAT to the mice diet) and high-dose PMEAT (200 mg/kg PMEAT addition) treatments in STZ-induced diabetic mice. After 6 weeks of intervention, significantly decreased fasting blood glucose (FBG) (*p* < 0.05), significantly decreased area under the curve (AUC) of the oral glucose tolerance test (*p* < 0.05), significantly decreased HOMA-IR (*p* < 0.05), and significantly increased HOMA-β (*p* < 0.05) were observed in the high-dose PMEAT group. Moreover, we performed an antioxidant activity experiment *in vitro*. The results showed that PMEAT had a strong ability to scavenge DPPH (IC_50_, 0.044 mg/mL) as well as ABTS free radicals (IC_50_, 0.040 mg/mL). In an animal experiment conducted on oxidative damage mice model which was induced by D-glucose and a high-fat diet, we observed significantly increased dismutase (SOD) (*p* < 0.01), glutathione (GSH) (*p* < 0.01), and glutathione peroxidase (GSH-Px) (*p* < 0.01) and significantly reduced malondialdehyde (MDA) and 8-ISO-prostaglandin-PGF2α (8-ISO-PGF2α), after treatment with PMEAT for 90 days. In conclusion, this study reveals the therapeutic potential of *Amomum tsao-ko* for the treatment of diabetes and helps us discover new antioxidant candidates from natural sources.

## Introduction

In the last three decades, especially high-calorie diets have been shown to affect lipid metabolism and lead to fat accumulation, resulting in obesity, which is a cause of various chronic diseases such as diabetes, hypertension, and cardiovascular disease (1). Many bioactive phytochemicals in natural plants have attracted the attention of researchers due to their hypoglycemic, lipid- lowering, and antioxidant properties (2, 3). Screening the safe, effective, and conveniently extracted compounds from natural plants have become a new direction to managing diabetes and alleviating oxidative damage.

*Amomum tsao-ko* Crevost et Lemaire (*A. tsao-ko*), a perennial plant classified into the Zingiberaceae family, is a medicinal herb discovered in Southeast Asia. In China, it is mainly distributed in the Guangxi, Guizhou, and Yunnan provinces. *A. tsao-ko* can not only be used as an edible spice but also has therapeutic effects on stomach disorders, dyspepsia, nausea, abdominal pain, and throat infections, as recorded in the *Chinese Pharmacopoeia*. Flavonoids, coumarins, phenols, and diterpenoids were found as bioactive constituents in *A. tsao-ko* extracts, which have been reported to have the activity of anti-obesity (4), bone health promotion (5), anti-inflammation (6), and anti-proliferation (7). He et al. found that amomutsaokols A-K, diarylheptanoids, tsaokopyranols A–M, and 2,6-epoxy diarylheptanoids from a 50% ethanol-water extract of *A. tsao-ko* demonstrated significant α-glucosidase inhibitory activity (8, 9). However, very limited studies have reported the hypoglycemic and antioxidant effects of methanol extracts from *A. tsao-ko*.

Over the past few years, we have carried out systematic research on *A. tsao-ko*. At the earliest, we did animal experiments with 12 kinds of spices (*A. tsao-ko*, star anise, cinnamon, etc.) to study their physiological regulating activities (10). The results indicated that four of them had the effects of reducing body weight and blood lipids, among which the activity of *A. tsao-ko* was the strongest. Next, we added 0.5 and 0.1% of the above four spices to the mouse feed to study if low-dose added spices have a biological effect on mice (11). The results showed that *A. tsao-ko*, aniseed, and cumin had hypoglycemic and hypolipidemic effects when 0.5% was added. When the addition amount was 0.1% (equivalent to 0.5 g/d), only *A. tsao-ko* exhibited hypoglycemic prosperity. Based on the above studies, the activity of the residue, acetone extract, chloroform extract, and methanol extract of *A. tsao-ko* were, respectively, assessed by performing animal experiments (12, 13). The results showed that the effective components of weight loss, lipid reduction, and glucose reduction mainly existed in the methanol extract. Because methanol is toxic, we explored the feasibility of using ethanol solution as a nontoxic solvent instead of a methanol-chloroform mixed solution (14). The results did not show that the ethanol extract had the effects of reducing blood lipids and regulating glucose homeostasis. The reason is probably that the phytochemicals in the *A. tsao-ko* were polar lipid solubles; nevertheless, the introduction of water not only changed the polarity of the solution but also altered the extraction components.

The present study was conducted as a further analysis to identify the bioactive constituents contained in the methanol extracts of *A. tsao-ko*. Because oxidative stress can lead to disorders of glucose metabolism, we currently performed a comprehensive and systematic study on the hypoglycemic and antioxidant activities of the power of methanol extracts of *A. tsao-ko* (PMEAT), *in vivo* and *in vitro*. This study may provide important evidence for the utilization and pharmaceutical development of methanol extracts of *A. tsao-ko*.

## Materials and Methods

### Chemicals

α-Glucosidase Detection Kit was purchased from Shanghai Guyan Industrial Co., Ltd. (Shanghai, China). Acarbose standard sample was purchased from Shijiazhuang Huarong Pharmaceutical Co., Ltd. (Shijiazhuang, China). The Insulin ELISA kit was purchased from Shijiazhuang Huiduan Biotechnology Co., Ltd. (Shijiazhuang, China). Superoxide dismutase (SOD) assay kit, reduced glutathione (GSH) assay kit, glutathione peroxidase (GSH-Px) assay kit, malondialdehyde (MDA) assay kit, and mouse 8-iso prostaglandin F2α (8-ISO-PGF2α) ELISA kit were purchased from Nanjing Jiancheng Bioengineering Institute (Nanjing, China). Other reagents were all analytical grade.

### Plant Materials and Preparation of PMEAT

Dried *A. tsao-ko* fruits were purchased from Kunming herbal market in Yunnan province, China. We prepared PMEAT according to the methods of Yan et al. with slight modifications (15). First, *A. tsao-ko* fruits were shelled, freeze-dried, pulverized using a high-speed shredder (HC100, Zhejiang Jinhui Machinery Factory, Yongkang, China) and sieved (100 mesh). A total of 50 g fruit powder was added to the mixed alcohol extracting solution (200 mL chloroform, 400 mL methanol, and 40 mL distilled water) and stayed still for 12 h. After being mixed for 5 min (18,000 r/min), the mixed liquor was crushed for 10 min (70% power, 20–25 Hz) with an ultrasonic cell pulverizer (XD-1200D, Nanjing Xianou Instrument Manufacturing Co., Ltd., Nanjing, China). After being shrunk and filtered by vacuum, the crushed mixture was concentrated and turned into a filter cake, which was added with a half amount of the mixed ethanol extract (100 mL chloroform, 200 mL methanol, and 20 mL distilled water) to complete the first-time extraction. Next, extraction was repeated twice in the same way and the filtrate was collected from three extractions. A 60 g activated silica gel powder was then added to the filtrate and the powdery mixture was obtained after being concentrated using a rotary evaporator (RE-52AA, Shanghai Yarong Biochemical Instrument Factory, Shanghai, China). The powder sample was then put in a self-made silica gel hydrogen column (200–300 mesh, 500 × 250 mm, activated by methanol, acetone, and chloroform for 4 h) and eluted successively with 600 mL chloroform, 600 mL acetone, and 600 mL methanol. Thus, we obtained the methanol eluent, which was then filtered by a microporous membrane (0.22 μm). The powder of methanol extract of *A. tsao-ko* was finally obtained after the evaporative concentration and freeze-drying.

### Identification of Bioactive Compounds in PMEAT

Chromatographic separation was performed in a Thermo Ultimate 3000LC system equipped with a Zorbax Eclipse C18 column (1.8 μm × 2.1 × 100 mm) maintained at 30°C (16). The mobile phases were 0.1% formic acid in water (A) and grade acetonitrile (B). The injection volume of the sample was 2 μL and the elution flow rate was 0.30 mL/min. The program of gradient elution was as follows: 0–2 min, 5% B; 2–7 min, 30% B; 7–14 min, 78% B; 14–20 min, 90% B; 20–25 min, 5% B. The ESI-MSn experiments were conducted on a Thermo Q Exactive HF mass spectrometer with a spray voltage of 3.5 kV in positive and negative modes. The sheath gas, auxiliary gas, and sweep gas flow rates were set to 45, 15, and 1 arbitrary unit, respectively. The heater and capillary temperatures were 325 and 330°C, respectively. The S-Lens RF Level was 55%. The analyzer scanned over a mass range of m/z 100–1,500 for a full scan at a mass resolution of 12,000. Data-dependent secondary mass spectrometry scanning (dd-MS2, TopN = 10) was at a mass resolution of 6,000 with an HCD mode.

### Determination of α-Glucosidase Inhibiting Activity *in vitro*

The determination procedure of α-glucosidase (α-GIA) inhibiting activity was referred to as the method of Chen et al. (17). In brief, α-glucosidase and PNPG (4-N-trophenyl-α-D-glu-copyranoside) were dissolved in a phosphate buffer solution (PBS) (10 mg/mL, pH 6.8) at 1 U/mL and 12.5 mmol/L, respectively. In addition, PMEAT samples were dissolved in a PBS (10 mg/mL, pH 6.8) to prepare different reaction gradient solutions. Forty microliters each of PMEAT sample solution and α-glucosidase solution were mixed in a 96-well microplate and incubated at 37°C for 15 min. Then, 20 μL of the PNPG solution was added and incubated at 37°C for 15 min. Finally, the reaction was stopped by the addition of 100 μL of Na_2_CO_3_ solution (pH 6.8). PBS was taken as blank control and was used as a background solution instead of α-glucosidase solution to eliminate the influence of the ground effect. The optical density (OD) of each well was detected using a microplate reader at a wavelength of 405 nm. The inhibition rate against α-glucosidase of two test solutions was calculated as follows:


(1) $$\begin{array}{r} \text{I}\left(\%\right)=\frac{\text{OD}blank-\left(\text{OD}sample-\text{OD}ground\right)}{\text{ODblank}}\times 100\% \end{array}$$

### Determination of Hypoglycemic Activity *in vivo*

#### Animals

Eight-week-old male ICR mice (SPF, weight 28–32 g) [Animal license number: SCXK(Jing)2016-0006] were obtained from Beijing Vital River Laboratory Animal Technology Co., Ltd (Beijing, China). Animals were housed in a controlled environment (temperature 20–25°C, humidity 40–60%, 12/12h light/dark cycle) with free access to pellet diet and water. The mice were used for experiments after 1 week of acclimatization.

#### Animal Model and Experimental Design

First, mice were randomly divided into two groups: a control group (Con, *n* = 10), fed the basic mouse fodder (starch 47.8%, casein 20%, sucrose 15%, lard 6%, cellulose 5%, minerals 4%, vitamins 2%, and L-methionine 0.2%), and a high-fat diet group (*n* = 40), fed high-fat diet (HFD basic mouse fodder adding 10% lard, e.g., 25 g lard were added to 250 g basic mouse fodder). From the 7th week, the mice in the HFD group were injected intraperitoneally with 150 mg/kg·BW STZ (Sterptozotocin) solution (STZ was dissolved with 0.1 mol/L pH 4.5 citrate buffer) after fasting for 12 h. Seventy-two hours after injection and fasting for 12 h again, tail vein blood was taken to determine the fasting blood glucose (FBG) concentration of the mice. The mice in the HFD group with HBG levels ≥ 11.1 mmol/L were selected as diabetic mice (18, 19). Finally, a total of 30 mice in the HFD group were confirmed to be successfully established as diabetic mice, together with 10 mice remaining in the control group for the subsequent 6 weeks of experiments.

Thirty diabetic mice were then randomized into three groups (*n* = 10 per group) as follows: the model group (Mod), the low-dose PMEAT group (PMEAT-L), and the high-dose PMEAT group (PMEAT-H). From week 8 to week 13, the control group was fed with basic mouse fodder, the model group was fed with high-fat diet, the PMEAT-L group was fed with high-fat diet adding 100 mg/kg PMEAT, and the PMEAT-H group was fed with high-fat diet adding 200 mg/kg PMEAT (the study flow chart and design see Supplementary Figure 1). Animal identification used 5% picric acid. The food intake and drinking water of each group were recorded on the first day of every week. On the last day of the 7th and 13th week, mice were housed in metabolic cages for 24 h, and the volumes of food intake and water intake were measured. On the same day, weight was also measured for each mouse individually.

#### Assessment of Impaired Blood Fasting Glucose

The blood glucose levels of tail blood in four mice groups after a 6-h fasting period were measured once every 2 weeks, starting with the 8th week of the experiment (before the PMEAT intervention).

#### Oral Glucose Tolerance Test

The oral glucose tolerance tests were performed in week 8 (before the PMEAT intervention) and week 13 (after the PMEAT intervention) on every group. After 10 h of fasting, all mice were intraperitoneally injected with a glucose solution (1.5 g/kg·BW). The blood glucose levels of the tail tip were measured at 0 (baseline), 15, 30, 60, and 120 min after the injection, using a one-touch glucometer (UltraTM, American Johnson & Johnson Co., Ltd, New Brunswick, USA). The integrated glucose response (area under the curve, AUC) over 2 h after the glucose overload was calculated as follows (20):


(2) $$\begin{array}{l} AUC=[(BG_{0}+BG_{15})+(BG_{15}+BG_{30})+(BG_{30}+BG_{60})\times 2\\ \;+(BG_{60}+BG_{120})\times 4]\times 0.25\times \;0.5 \end{array}$$

BGi: blood glucose value at i min.

#### Assessment of Insulin Resistance

In the 13th week (after 6 weeks of PMEAT intervention), the blood of mice in four groups was collected from the retro-ocular venous after fasting for 10 h. The fasting blood glucose was measured with the OneTouch UltraTM rapid blood glucose meter. The remaining blood was centrifuged (3,000 r/min, 15 min) to collect plasma, and plasma insulin was quantified by an enzyme-linked immunosorbent assay (ELISA) kit. Homeostatic model assessment for insulin resistance (HOMA-IR) and homeostatic model assessment for β cell function (HOMA-β) were calculated based on fasting blood glucose (FBG) and fasting insulin level (21). Calculation formula: HOMA-IR = FBG (mmol/L) × fasting insulin level (μU/ml)/22.5; HOMA-β = [20 × fasting insulin level (mIU/L)]/[FBG (mmol/L)−3.5] (%).

### Determination of Antioxidant Activity *in vitro*

#### DPPH Radical Scavenging Assay

The DPPH assay was performed according to the method of Cui et al. with slight modifications (22). In brief, 0.3 mL of the diluted test sample and 3 mL 25 μg/mL of a DPPH working solution were added to the test tube, reacted at 30°C in the dark for 40 min, and measured at 516 nm. Methanol was the blank control and rutin and curcumin were the positive controls. The scavenging activity was expressed as an IC_50_ value (mg/mL).

#### ABTS Radical Scavenging Assay

The ABTS assay was undertaken by the method of Cui et al. with slight modifications (22). First, an ABTS stock solution was prepared by mixing 5 mL of 7 mmol/L ABTS methanol solution and 88 μL of 140 mmol/L potassium per sulfate solution at 25°C in the dark for 16 h. Methanol was used to dilute the stock solution for the absorbance to reach 0.7 ± 0.02 at 734 nm, then to get the ABTS working solution. Next, 0.3 mL of the diluted test sample and 3 mL ABTS working solution were added to the test tube to react at 30°C in the dark for 6 min, and the absorbance was subsequently measured at 734 nm. Methanol was the blank control and rutin and curcumin were the positive controls. The scavenging activity was expressed as an IC_50_ value (mg/mL).

#### Ferric Reducing Antioxidant Power Assay

The FRAP assay was performed using the method of Guo et al. (23). The FRAP reagent was prepared by mixing 300 mmol/L acetate buffer (pH 3.6), 10 mmol/L TPTZ, and 20 mmol/L FeCl_3_ at a ratio of 10:1:1 (v/v/v). Subsequently, 0.1 mL of the diluted test sample and 3 mL FRAP reagent were added to the test tube and reacted at 37°C in the dark for 5 min. The absorbance was measured at 596 nm. With the standard solution of FeSO_4_ as the reference substance, the FRAP result of the samples was presented as the millimoles of FeSO_4_ required to achieve the same absorbance.

### Determination of Antioxidant Activity *in vivo*

#### Animal Model and Experimental Design

To evaluate the antioxidant activity of PMEAT *in vivo*, we performed another 90-day animal experiment based on the oxidative damage mouse model (24). After 1 week of acclimation, mice (the strain and raising environment were the same as the mice in the hypoglycemic animal experiment) were randomly divided into four different groups: a control group (Con, *n* = 12), a model group (Mod, *n* = 12), a low-dose PMEAT group (PMEAT-L, *n* = 12), and a high-dose PMEAT group (PMEAT-H, *n* = 12). The mice in the control group were fed a basic diet for 8 weeks. The mice in the model group were fed a high-fat diet (HFD) for 8 weeks. The mice in the PMEAT-L and PMEAT-H groups were treated with HFD adding 100 mg/kg PMEAT and HFD adding 200 mg/kg PMEAT for 8 weeks, respectively. During this same 8-week period, the mice in each group except the control group were intraperitoneally injected with D-galucose (125 mg/kg·d) once per day (15). After the D-galactose injection was terminated, each group continued to be treated with its diet for another 5 weeks to complete the 90-day experiment plan (the study flow chart and design see Supplementary Figure 2).

At the end of the feeding experiment, all mice fasted for 24 h (not having food and water), then blood and liver tissues were collected after sacrificing (25, 26). Blood samples from all mice were spun for 15 min at 3,000 rpm. The plasma was then isolated and used to assess the levels of SOD, GSH, GSH-Px, MDA, and 8-ISO-PGF2α using appropriate test kits (16). The liver tissues were ground and spun for 20 min at 3,000 rpm to get a 10% homogenate. The supernatants were then collected, and the levels of SOD, GSH, GSH-Px, and MDA were assessed using the corresponding test kits (16).

### Statistical Analysis

Statistical analysis was performed using SPSS version 26.0. Data were tested for normal distribution with the Shapiro–Wilk test. One-way ANOVA followed by Tukey's-SHD and Student's *t*-test were performed to examine the differences among treatment groups for the analysis of the hypoglycemic indicators (fasting blood glucose, glucose tolerance AUC, HOMA-IR, and HOMA-β) and antioxidant indices (plasma SOD, plasma GSM, plasma GSH-Px, plasma MDA, plasma 8-ISO-PGF2α, liver tissue SOD, liver tissue GSM, liver tissue GSH-Px, and liver tissue MDA). *P*-value < 0.05 indicated that the difference between groups was statistically significant.

## Results

### Bioactive Constituents Identified in PMEAT

**Figure 1:**
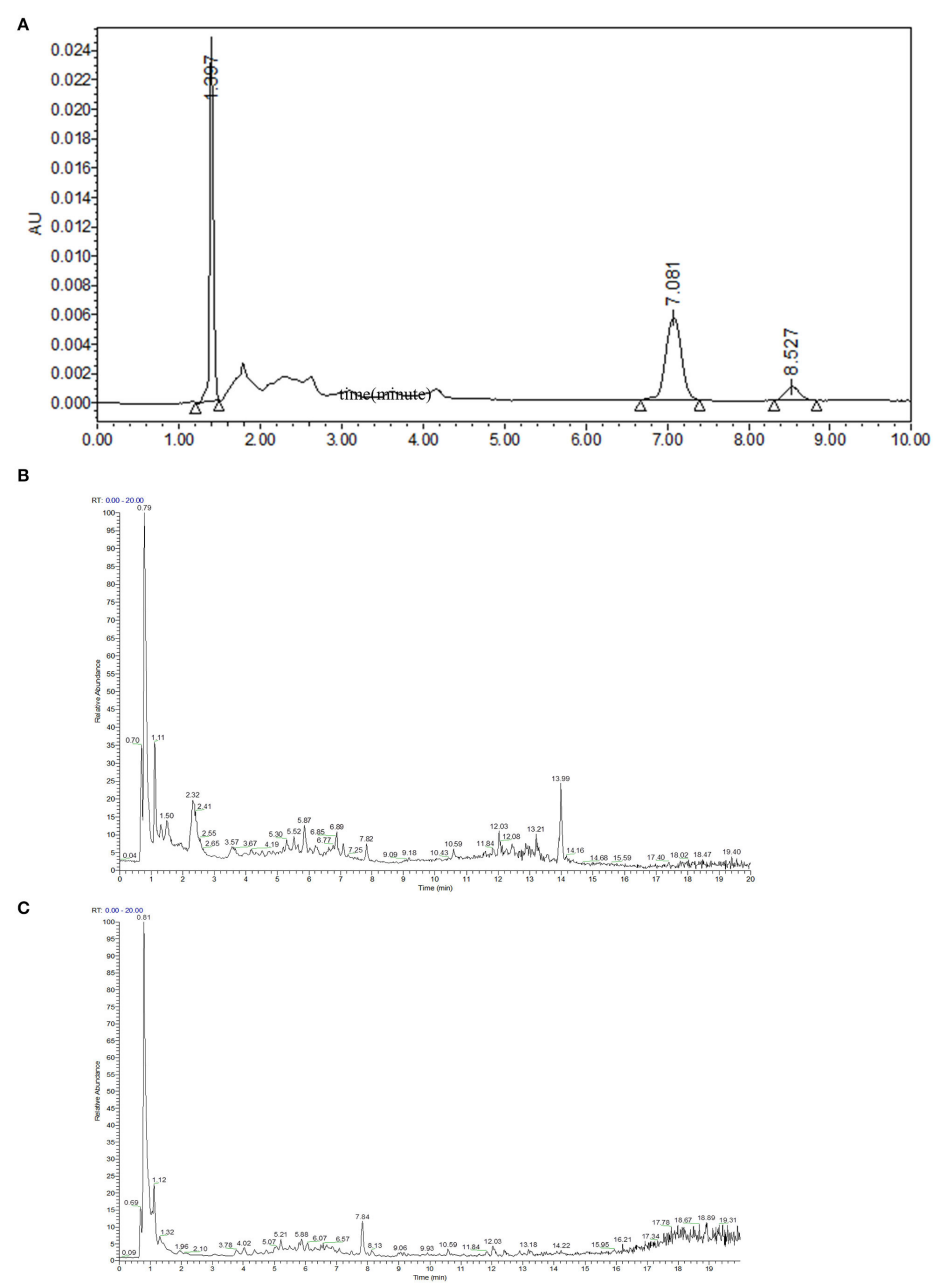
Separation and identification of phytochemicals in PMEAT by HPLC-MS/MS. **(A)** The HPLC analysis chart. **(B,C)** The base peak chromatogram (BPC) for phytochemicals in *A. tsao-ko* methanol extract in the ESI+ **(B)** and ESI– **(C)** modes.

To elucidate the substance basis of the pharmacologic effect, a HPLC-MS/MS full-spectrum analysis in positive and negative ionization modes was performed (Figure 1). The fragment information was compared with the Thermo mzCloud and Thermo mzValu databases. The phytochemicals in the PMEAT were summarized and displayed in Table 1. Twenty-one flavonoids including 8-prenylnaringenin (No 3), dihydrokaempferol (No 6), isorhamnetin 3-alactoside (No 8), avicularin (No 11), epicatechin (No 12), hesperetin (No 13), hyperoside (No 14), isorhamnetin (No 15), padmatin (No 18), procyanidin A2 (No 19), quercetin (No 20), rutin (No 21), ambocin (No 23), isorhamnetin-3-O-glucoside (No 26), isorhamnetin-3-O-rutinoside (No 27), naringin (No 28), procyanidin B1 (No 29), astragalin (No 31), catechin (No 32), luteolin (No 34), and quercetin-3β-D-glucoside (No 35) were identified. Four coumarins including 5,6,7-trimethoxycoumarin (No 1), 6-methylcoumarin (No 2), 4-hydroxycoumarin (No 10), and isofraxidin (No 25) were identified. Three phenols were identified in PMEAT, namely gentisyl alcohol (No 7), metoprolol analogs (No 16), and vanillin (No 22). Eight other phytochemicals were identified, namely atenolol analogs (No 4), coniferaldehyde (No 5), 2'-O-methyladenosine (No 9), oxprenolol (No 17), atenolol acid analogs (No 24), vanillic acid (No 30), ferulic acid (No 33), and α,α-trehalose (No 36).

**Table 1:** Identification of phytochemicals in PMEAT by HPLC-MS/MS.

**No**	**RT (min)**	**Calc. MW**	**Annotation MW**	**Adducts**	**Annot. DeltaMass (ppm)**	**Formula**	**Identification**	**Activity**
1	9.038	236.06815	236.06847	(M + H)+	−1.38	C_12_ H_12_ O_5_	5,6,7-Trimethoxycoumarin	Antioxidant (41)
2	7.484	160.05234	160.05243	(M + H)+	−0.59	C_10_ H_8_ O_2_	6-Methylcoumarin	Antioxidant (42)
3	10.344	340.13069	340.13107	(M + H)+	−1.12	C_20_ H_20_ O_5_	8-Prenylnaringenin	Monoacylglycerol lipase inhibitor (43)
4	6.306	266.16283	266.16304	(M + H)+	−0.8	C_14_ H_22_ N_2_ O_3_	Atenolol	β-Blocker (44)
5	10.072	178.06302	178.06299	(M + H)+	0.15	C_10_ H_10_ O_3_	Coniferaldehyde	Reduce intracellular triglyceride (45)
6	5.937	288.06319	288.06339	(M + H)+	−0.69	C_15_ H_12_ O_6_	Dihydrokaempferol	Antioxidant (46)
7	6.029	140.04734	140.04734	(M + H)+	−0.05	C_7_ H_8_ O_3_	Gentisyl alcohol	Antioxidant (47)
8	7.075	478.11098	478.11113	(M + H)+	−0.31	C_22_ H_22_ O_12_	Isorhamnetin 3-galactoside	Plasminogen activator inhibitor (48);DPP-IV inhibitor (54)
9	1.69	281.11223	281.1124	(M + H)+	−0.62	C_11_ H_15_ N_5_ O_4_	2'-O-Methyladenosine	–
10	12.219	162.03156	162.03169	(M + H)+	−0.85	C_9_ H_6_ O_3_	4-Hydroxycoumarin	α-Glucosidase inhibitor (49)
11	6.967	434.08455	434.08491	(M + H)+	−0.83	C_20_ H_18_ O_11_	Avicularin	α-Glucosidase inhibitor (50)
12	5.877	290.07881	290.07904	(M + H)+	−0.77	C_15_ H_14_ O_6_	Epicatechin	α-Glucosidase inhibitor (51); Antioxidant (52)
13	5.085	302.07875	302.07904	(M + H)+	−0.95	C_16_ H_14_ O_6_	Hesperetin	α-Glucosidase inhibitor (51); Antioxidant (53)
14	6.676	464.09545	464.09548	(M + H)+	−0.07	C_21_ H_20_ O_12_	Hyperoside	Antioxidant (55)
15	7.073	316.05791	316.0583	(M + H)+	−1.25	C_16_ H_12_ O_7_	Isorhamnetin	α-Glucosidase inhibitor (54)
16	4.897	267.18324	267.18344	(M + H)+	−0.74	C_15_ H_25_ N O_3_	Metoprolol	β-Blocker (44)
17	2.338	265.16756	265.16779	(M + H)+	−0.89	C_15_ H_23_ N O_3_	Oxprenolol	β-Blocker (44)
18	7.493	318.07356	318.07395	(M + H)+	−1.22	C_16_ H_14_ O_7_	Padmatin	-
19	7.921	576.12645	576.12678	(M + H)+	−0.56	C_30_ H_24_ O_12_	Procyanidin A2	Antioxidant (56)
20	6.675	302.04247	302.04265	(M + H)+	−0.61	C_15_ H_10_ O_7_	Quercetin	α-Glucosidase inhibitor (50)
21	6.456	610.15318	610.15338	(M + H)+	−0.33	C_27_ H_30_ O_16_	Rutin	Antioxidant (53)
22	6.556	152.04735	152.04734	(M + H)+	0.04	C_8_ H_8_ O_3_	Vanillin	α-Glucosidase inhibitor (57); antioxidant (58)
23	6.969	564.14833	564.14791	(M – H) –	0.75	C_26_ H_28_ O_14_	Ambocin	-
24	6.384	267.14717	267.14706	(M – H) –	0.42	C_14_ H_21_ N O_4_	Atenolol acid	β-Blocker (44)
25	5.752	222.05253	222.05282	(M – H) –	−1.3	C_11_ H_10_ O_5_	Isofraxidin	NF-_*k*_B inhibitor (59); antioxidant (59)
26	7.075	478.11137	478.11113	(M – H) –	0.52	C_22_ H_22_ O_12_	isorhamnetin-3-O-glucoside	α-Glucosidase inhibitor (54)
27	6.857	624.16969	624.16903	(M – H) –	1.05	C_28_ H_32_ O_16_	isorhamnetin-3-O-rutinoside	α-Glucosidase inhibitor (54)
28	6.729	580.17976	580.17921	(M – H) –	0.96	C_27_ H_32_ O_14_	Naringin	α-Glucosidase inhibitor (60); antioxidant (60)
29	5.623	578.14284	578.14243	(M – H) –	0.71	C_30_ H_26_ O_12_	Procyanidin B1	α-Glucosidase inhibitor (51); aldose reductase inhibitor (61); antioxidant (52)
30	5.497	168.04149	168.04226	(M – H) –	−4.59	C_8_ H_8_ O_4_	Vanillic acid	Antioxidant (58)
31	7.418	448.101	448.10056	(M – H) –	0.97	C_21_ H_20_ O_11_	Astragalin	Antioxidant (62)
32	5.354	290.07915	290.07904	(M – H) –	0.37	C_15_ H_14_ O_6_	Catechin	Antioxidant (12)
33	6.965	194.05736	194.05791	(M – H) –	−2.85	C_10_ H_10_ O_4_	Ferulic acid	Antioxidant (18)
34	9.849	286.04777	286.04774	(M – H) –	0.12	C_15_ H_10_ O_6_	Luteolin	α-Glucosidase inhibitor (14)
35	6.677	464.096	464.09548	(M – H) –	1.12	C_21_ H_20_ O_12_	Quercetin-3β-D-glucoside	α-Glucosidase inhibitor (51); antioxidant (21)
36	0.8	342.11661	342.11621	(M – H) –	1.16	C_12_ H_22_ O_11_	α,α-Trehalose	Antioxidant (29)

Based on the content data of the constituents and the findings, we inferred that phenols, flavonoids, and oligosaccharides were probably the main compounds that exhibited the hypoglycemic and antioxidant activities (27, 28). Among the above 36 phytochemicals, in particular, the α,α-trehalose in PMEAT, a natural non-reducing disaccharide, has been reported to possess a pleiotropic role in various physiological conditions such as oxidative stress protection, inflammation prevention, and inhibition of protein denaturation and neurodegeneration (29). According to the quantitative analysis, its content proportion in PMEAT was not <13%. These findings suggest that the antioxidant and hypoglycemic effects of *A. tsao-ko* may be mainly attributed to this category of phytochemicals.

### Inhibitory Activity Against α-Glucosidase *in vitro*

**Figure 2:**
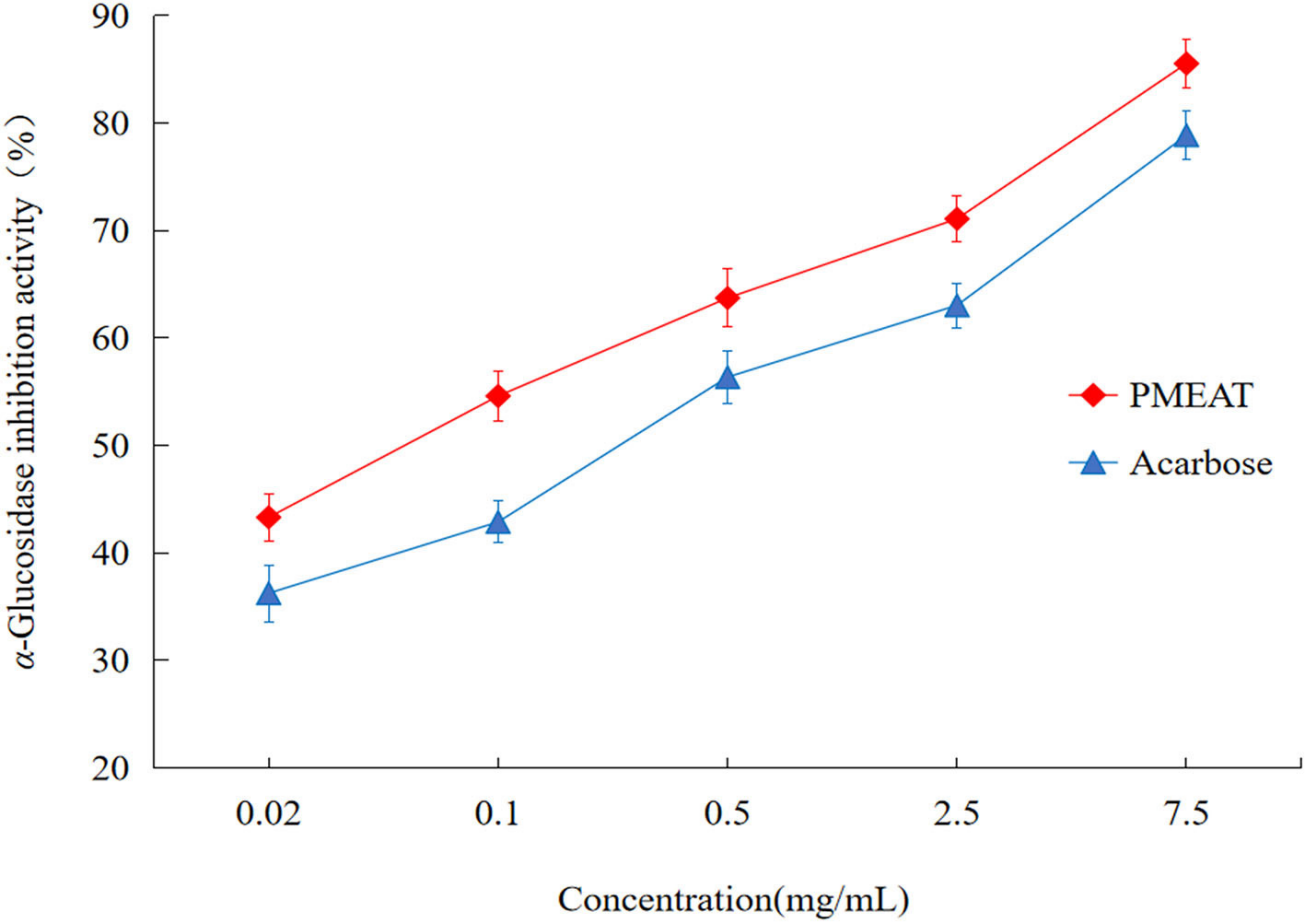
The *in vitro* α*-*glucosidase inhibitory activity of PMEAT. The line chart depicting the inhibition rate against α-glucosidase of PMEAT (red color) and acarbose (blue color) at different solution concentrations. Three parallel samples for the two test solutions were used to perform this *in vitro* experiment.

To assess the *in vitro* hypoglycemic effect of PMEAT, α-GIA was investigated (Figure 2). We observed inhibition against α-glucosidase by PMEAT and acarbose in a dose-dependent relationship. Compared with the positive control (acarbose, IC_50_ 0.273 mg/mL), PMEAT had a higher α-GIA (IC_50_ 0.154 mg/mL). When the concentration of the PMEAT solution was 0.5 mg/mL, the inhibition rate reached 63.72%. The above strong inhibitory activity against α-glucosidase was probably attributed to a variety of phenolic and coumarins compounds in PMEAT. Many researchers have also confirmed that 4-Hydroxycoumarin, Avicularin, Epicatechin, Hesperetin, Quercetin, Procyanidin B1, and Quercetin-3β-D-glucoside have varying degrees of α-glucosidase inhabitation activities (49–51).

### Hypoglycemic Activity of PMEAT *in vivo*

**Figure 3:**
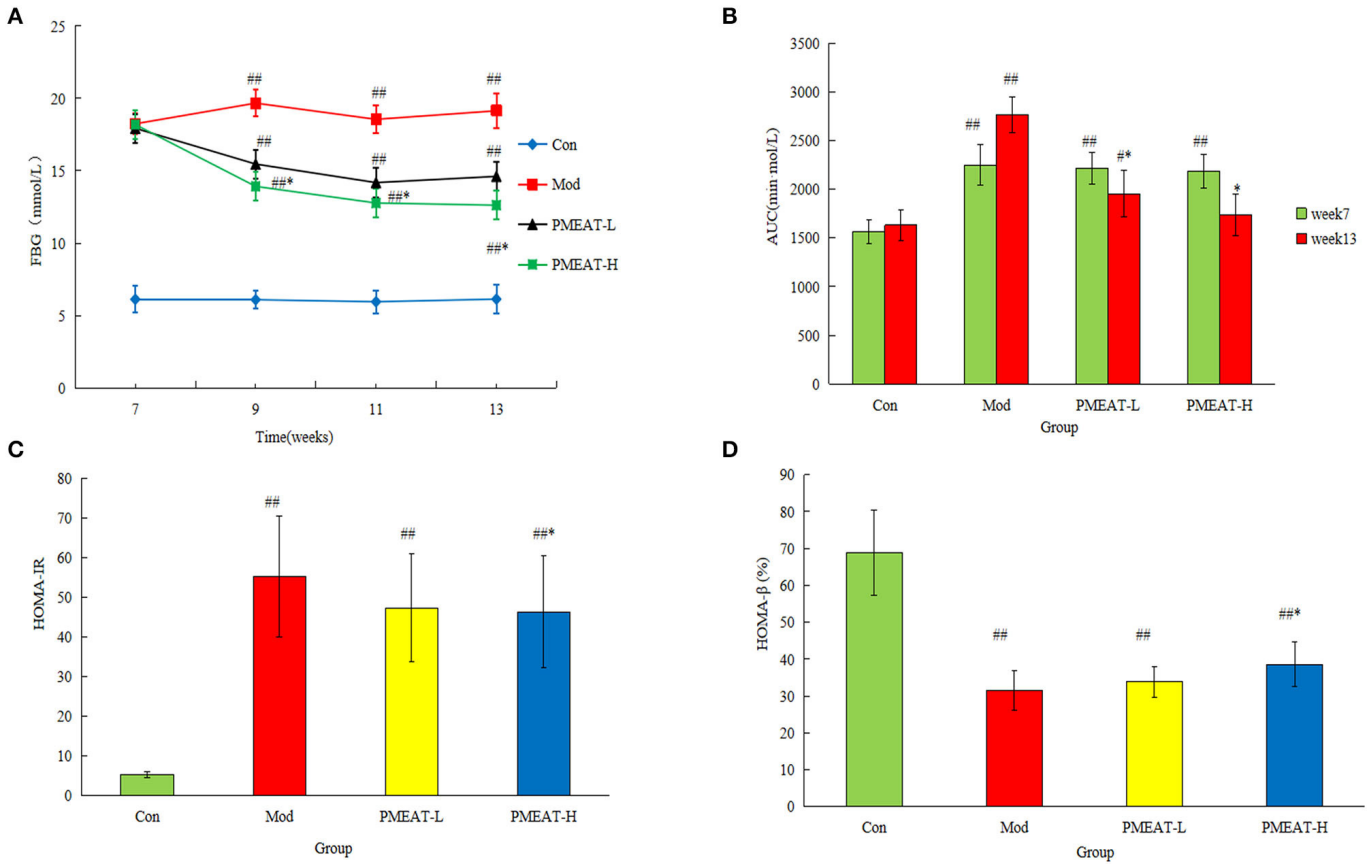
The hypoglycemic effect of PMEAT on STZ-induced diabetic mice. **(A)** Fasting blood glucose of four observed groups at different time points. **(B)** AUC of four observed groups. **(C)** HOMA-IR of four observed groups. **(D)** HOMA-β of four observed groups. One-way ANOVA was applied to examine the differences among four groups. Superscript ^#^ and ^##^ indicate statistically significant differences compared with the Con group (*p* < 0.05) and (*p* < 0.01), respectively. Superscript * indicate statistically significant differences compared with the Mod group (*p* < 0.05). Con, control group; Mod, model group; PMEAT-L, low-dose PMEAT group; PMEAT-H, high-dose PMEAT group.

The results of FBG are shown in Figure 3A. After the PMEAT treatment for 6 weeks, the FBG level in the PMEAT-H group was lower than that in the Mod group (*p* < 0.05). No significant difference was observed in PMEAT-L group when compared with the Mod group. As shown in Figure 3B, after 6 weeks of intervention with PMEAT, the AUC of the PMEAT-L group and the PMEAT-H group was lower than that in the Mod group (*p* < 0.05). The HOMA-IR of the PMEAT-H group was significantly (*p* < 0.05) lower than that in the Mod group as shown in Figure 3C. Moreover, the HOMA-β of the PMEAT-H group was significantly (*p* < 0.05) higher than that in the Mod group presented in Figure 3D. There are many ways of the mechanisms of hypoglycemic effect in the human body. Without mechanism research performed in this study, we could only infer that it was the contents of avicularin, epicatechin, hesperetin, and other components listed in Table 1 with α-glucosidase inhibitory activity in the PMEAT which contributed to the *in vivo* anti-diabetic properties. The compounds probably prevented oligosaccharides in food from decomposing into monosaccharides, and thus reducing the glucose absorption in the intestine and protected islet cells, and consequently enhanced glucose tolerance (30, 31).

### Antioxidant Activity of PMEAT *in vitro*

**Figure 4:**
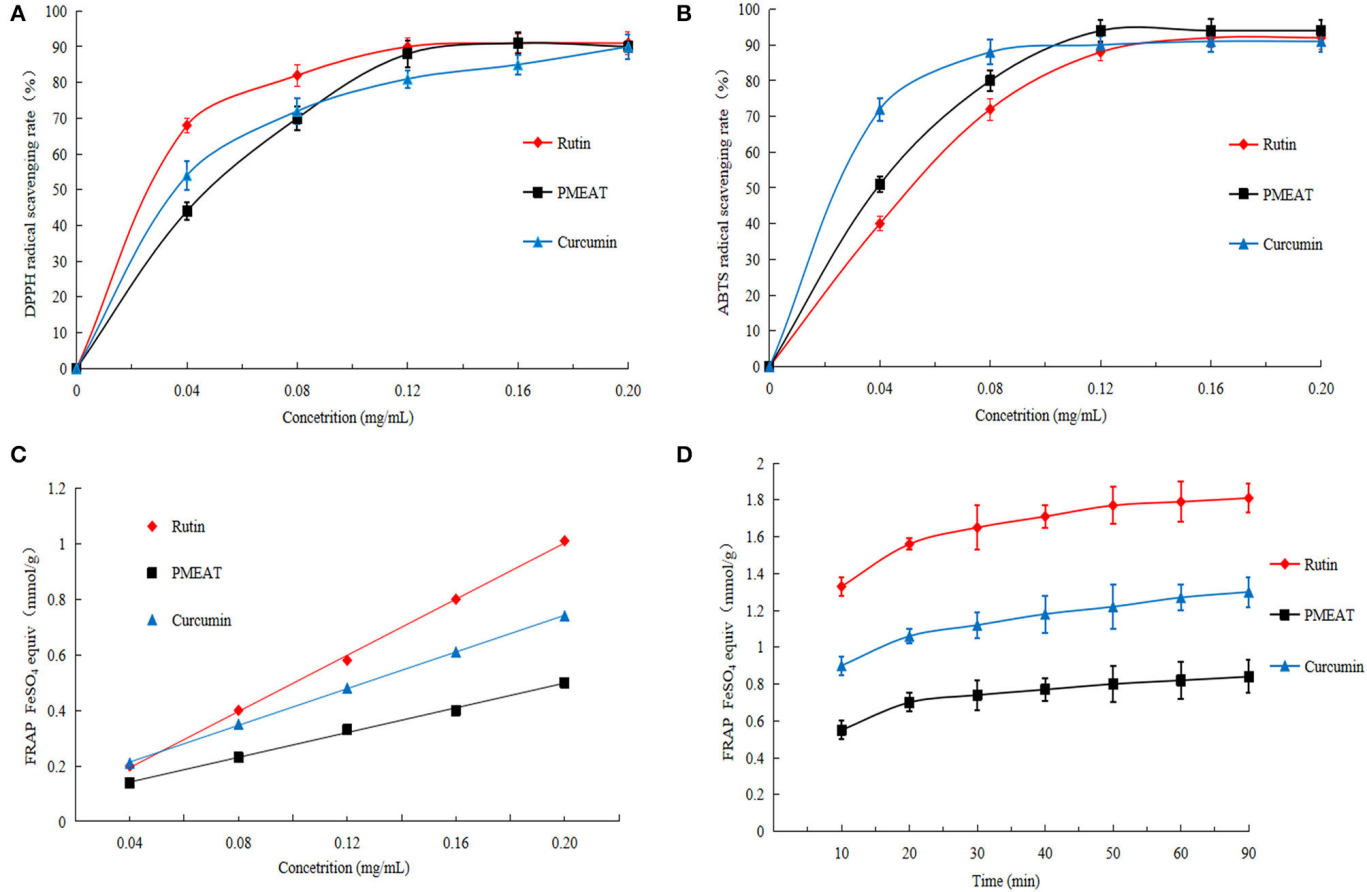
The *in vitro* antioxidant activity of PMEAT. **(A)** DPPH scavenging effect of rutin, PMEAT, and curcumin. **(B)** ABTS scavenging effect of rutin, PMEAT, and curcumin. **(C)** Result of FRAP assay of rutin, PMEAT, and curcumin. **(D)** FRAP time-dependent curve of rutin, PMEAT, and curcumin.

In this article, various assays (DPPH, ABTS, and FRAP) were used to comprehensively evaluate the antioxidant activities of *A. tsao-ko* methanol extracts. As shown in Figure 4A, when the concentration of PMEAT was higher than 0.08 mg/mL, the scavenging effect of PMEAT on DPPH free radicals was stronger than that of curcumin. The IC_50_ of DPPH scavenging of PMEAT was 0.044 mg/mL. Moreover, the scavenging rate curve of PMEAT coincided with the curve of rutin when the solution concentration increased to 0.12 mg/mL. The result of the ABTS radical scavenging of PMEAT is presented in Figure 4B. It shows that PMEAT had stronger activity against ABTS free radicals than rutin and curcumin when the concentration increased to 0.12 mg/mL. The IC_50_ was 0.04 mg/mL. FRAP assay is an indirect method based on a redox reaction to represent the antioxidant capacity of a test sample. The linear relationship between absorbance and FeSO_4_ concentrations produced a significant straight line (*Y* = *0.674X* + *0.0511, R*^2^ = *0.9996*). As shown in Figures 4C,D, the FRAP values of the three tested substances increased as the concentrations increased, and showed a time-dependent feature. However, the FRAP values of PMEAT were lower than that of rutin and curcumin at the same concentration and same time point. This indicates that the methanol extract of *A. tsao-ko* is inferior to rutin and curcumin in total antioxidant capacity.

It was probably due to α, α-trehalose, and procyanidin A2 identified in the PMEAT led to the *in vitro* antioxidant activity. As explicated in a review article by Chaitanya et al. (29), trehalose possessed an antioxidant activity by targeting cell progression, angiogenesis, and metastasis pathways at the molecular level. In addition, Wang et al. (56) reported that procyanidin A2 protected cells against the damage from inflammation and oxidative injury by targeting NF-kB, MAPK, and Nrf2 pathways in RAW264.7 cells. As a mixture of natural ingredients, PMEAT contained plenty of components that belonged to different categories and had complex structures, and thus its total antioxidant capacity was lower than that of the standard samples (rutin and curcumin).

### Antioxidant Activity of PMEAT *in vivo*

**Figure 5:**
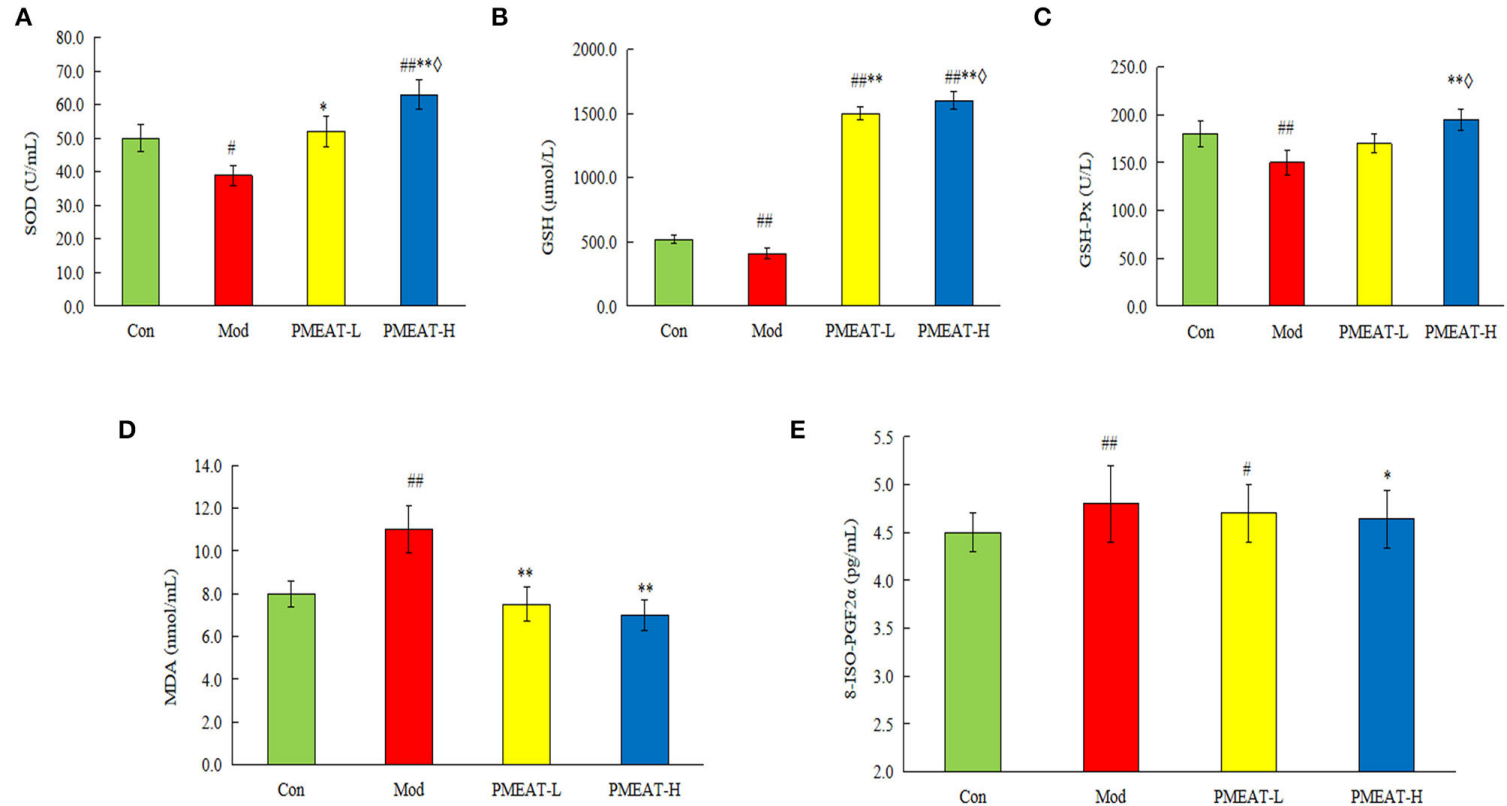
Effect of PMEAT treatment on plasma SOD, GSH, GSH-Px, MDA, and 8-ISO-PGF2α. **(A)** Plasma SOD of four observed groups. **(B)** Plasma GSH of four observed groups. **(C)** Plasma GSH-Px of four observed groups. **(D)** Plasma MDA of four observed groups. **(E)** Plasma 8-ISO-PGF2α of four observed groups. One-way ANOVA was applied to examine the differences among the three groups. Superscript ^#^ and ^##^ indicate statistically significant differences compared with the Con group (*p* < 0.05) and (*p* < 0.01), respectively. Superscript * and ** indicate statistically significant differences compared with the Mod group (*p* < 0.05) and (*p* < 0.01), respectively. Superscript ♢ indicates a statistically significant difference compared with the PMEAT-L group (*p* < 0.05). Con, control group; Mod, model group; PMEAT-L, low-dose PMEAT group; PMEAT-H, high-dose PMEAT group.

**Figure 6:**
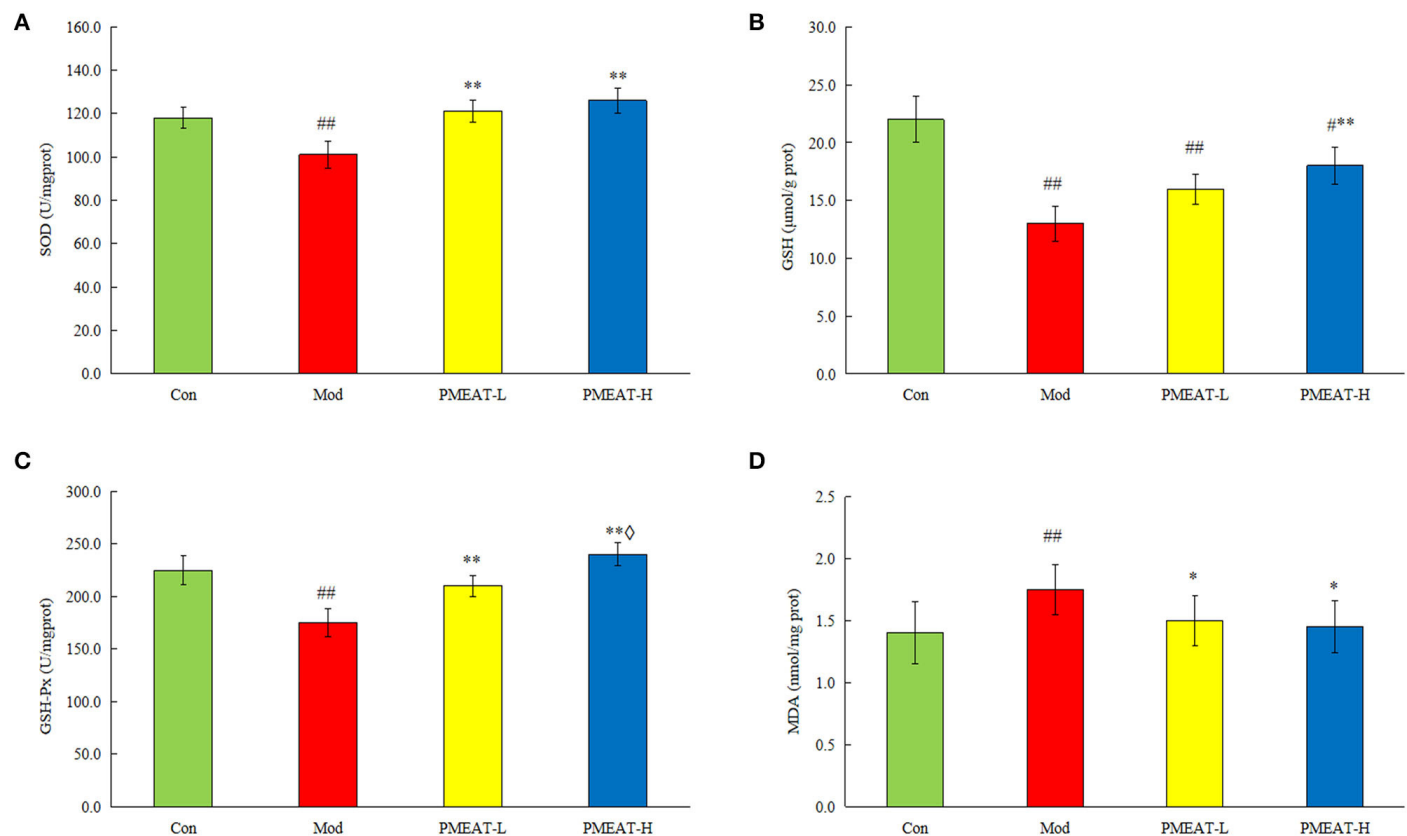
Effect of PMEAT treatment on liver tissue SOD, GSH, GSH-Px, MDA, and 8-ISO-PGF2α. **(A)** Concentration of SOD in liver tissue of four observed groups. **(B)** Concentration of GSH in liver tissue of four observed groups. **(C)** Concentration of GSH-Px in liver tissue of four observed groups. **(D)** Concentration of MDA in liver tissue of four observed groups. One-way ANOVA was applied to examine the differences among four groups. Superscript ^#^ and ^##^ indicate statistically significant differences compared with the Con group (*p* < 0.05) and (*p* < 0.01), respectively. Superscript * and ** indicate statistically significant differences compared with the Mod group (*p* < 0.05) and (*p* < 0.01), respectively. Superscript ♢ indicates a statistically significant difference compared with the PMEAT-L group (*p* < 0.05). Con, control group; Mod, model group; PMEAT-L, low-dose PMEAT group; PMEAT-H, high-dose PMEAT group.

As shown in Figure 5, compared with the control group, the mice in the model group induced by D-galactose plus HFD showed a statistically significant decrease in the levels of SOD, GSH, and GSH-Px in plasma. On the other hand, levels of MDA and 8-ISO-PGF2α in the model group were significantly higher than that in the control group. However, the addition of PMEAT to the diet more obviously reversed these biochemical abnormalities in plasma after the oxidative damage. These antioxidant effects were dose-dependent, which means the PMEAT-H group was more effective than the PMEAT-L group in terms of the indices of SOD, GSH, and GSH-Px in plasma. As presented in Figure 6, similar antioxidant effects were seen for the indices of SOD, GSH, GSH-Px, and MDA in liver tissue. Many studies (52, 53) have proved that the natural plants which contained flavonoids, phenols, and other ingredients with definite antioxidant effects would generally exhibit a good repairing effect on oxidative damage *in vivo*.

## Discussion

As a traditional Chinese medicine, *A. tsao-ko* has been studied by a few researchers at home and abroad (4–9). Using water extraction, ethanol extraction, and petroleum ether extraction, Liu et al. performed a comprehensive chemical investigation and found that the peel and the seeds of *A. tsao-ko* contained a variety of chemical components, including saccharide, protein, amino acids, phenolic compounds, tannins, organic acids, saponins, flavonoids, anthraquinone, coumarin, lactones, steroids, terpenoids, volatile oil, anthocyanins, and so on (32). Based on the previous results of animal experiments conducted with extracts by different extracting solvents, this study focused on the methanol extracts of *A. tsao-ko*, which were, nevertheless, seldom studied by other researchers. High-performance liquid chromatography with a mass spectrometry analysis revealed a total of 36 chemical components in PMEAT. To our knowledge, this is the first time the chemicals in the methanol extracts from the *A. tsao-ko* were isolated and qualitatively analyzed.

An accumulating body of evidence has shown that diabetic patients suffer from manifested oxidative stress due to hyperglycemia, hyperinsulinemia, and insulin resistance (33). Oxidative stress may bring about a variety of adverse effects to diabetes mellitus patients, such as endodermis cell injury and diabetic nephropathy. As a strategy to counteract the negative effect of oxidative stress, antioxidant-based therapy is becoming a promising way to minimize the complications associated with oxidative stress (34). This is why we examined both the anti-diabetic and the antioxidant effects of PMEAT in this study.

We evaluated the hypoglycemic activity of *A. tsao-ko in vitro* by an α-glucosidase assay. The IC_50_ of PMEAT on α-glucosidase was 0.145 mg/mL, which was lower than that of acarbose. Zhang et al. also investigated the hypoglycemic potential of methanol extract from *A. tsao-ko* by α-glucosidase assay and reported that the IC_50_ of α-glucosidase was 1.76 mg/mL (35). Under the action of α-glucosidase, glucose is released from the carbohydrates in food and enters the blood through the intestine, thereby leading to the increase of postprandial blood glucose. By slowing down the decomposition of starch into glucose, an α-glucosidase inhibitor delays or inhibits the absorption of glucose, and thus effectively reduces the postprandial hyperglycemia (36).

When blood glucose rises, appropriate amounts of insulin would be secreted from β cells to regulate the balance among muscle glycogen, liver glycogen, and blood glucose levels. In the current study, because of the cytotoxic effects of STZ on β cells, diabetic mice showed the symptoms of polyphagia, polyuria, polydipsia, and a severe loss in body weight. After the intervention with PMEAT for 6 weeks, the hyperglycemia, FBG, impaired glucose tolerance, and insulin resistance in mice were effectively improved, respectively. A similar finding was seen in the study performed by He et al. (37).

As many studies reported, polyphenols exhibited α-GIA through binding to α-glucosidase molecules. Polyphenols contain at least one aromatic ring with one or more hydroxyl groups in addition to other substituents. The interaction forces between polyphenols and α-glucosidase are expected to include hydrogen bonding and hydrophobic force. Electron delocalization between C=C (or C=O) and aromatic rings in polyphenols has been reported to enhance hydrophobic (π-π) interactions with α-glucosidase (38). Flavonoids, present in a wide variety of plant extracts, are the main substances in the polyphenols family. Its structure is represented by a benzene ring, condensed with a heterocyclic six-membered pyran or pyrone ring, which carries a phenyl ring in the second or third position as a substituent. In our study, 21 kinds of chemicals that belonged to flavonoids were identified in PMEAT (Table 1). Therefore, we speculate that the above *in vitro* and *in vivo* anti-diabetic effects of *A. tsao-ko* may be attributed to the biological activities of the flavonoid components.

Rutin and curcumin are often used as positive controls for the assessment of free radical scavenging capacity. In this study, the antioxidant capacity was evaluated from the perspectives of DPPH and ABTS free radical scavenging capacity. We found that the scavenging effect of DPPH and ABTS free radicals of PMEAT was inferior to that of curcumin at low concentrations but stronger than that of curcumin at high concentrations. The scavenging capacity of PMEAT on DPPH was weaker than that of rutin, but the scavenging effect on ABTS free radicals was stronger than that of rutin. These experimental results may be because the methanol extract from *A. tsao-ko* was a complex mixture and could not exhibit obvious antioxidant capacity at low concentrations. The antioxidant capacity increased considerably when the PMEAT solution reached a higher active concentration. This is consistent with the results of our previous studies (12, 13).

In *in vivo* study, an oxidative damage model of mice was established by D-galactose combined with a high-fat diet. Herein, we found that PMEAT treatment restored SOD, GSH-Px, and 8-ISO-PGF2α levels in the mice administered D-galactose to levels closer to or higher than those in the healthy mice. These results suggest that PMEAT can mitigate oxidative damage *in vivo*.

About the components associated with the antioxidant activity in PMEAT, we think that polyphenols and their derivatives, polysaccharides, and coumarins probably played the regulating role. Consistently, Yuan et al. studied the flavonoids extract of *A. tsao-ko* and established that they had strong DPPH free radical scavenging ability (39). Li et al. found in the *in vitro* experiment that polyphenols extracted from *A. tsao-ko* exhibited DPPH and ABTS free radical scavenging ability (40). Anti-oxidation is an important property of polyphenols. The ortho-phenolic hydroxyl in the phenolic hydroxyl structure (catechol or pyrogallol) can be easily oxidized, thereby making polyphenols have a strong ability to capture free radicals as well as reactive oxygen species. In addition, multiple ortho-phenolic hydroxyls in polyphenols can be utilized as a poly-ligand to act with metal ions, thereby forming stable five-ring chelates. By making the metal ions inactive in this way, polyphenol molecules effectively regulate the oxidation process. Nevertheless, the specific bioactive compounds correlated to the biological effects still need further research.

## Conclusion

Flavonoids, phenols, coumarins, oligosaccharides, and other bioactive constituents were identified in methanol extracts from *A. tsao-ko*. We confirmed the hypoglycemic potential of *A. tsao-ko* methanol extracts by α-glucosidase inhibition assay *in vitro* and STZ-induced mice intervention experiments *in vivo*. In addition, we verified the antioxidant activity of methanol extracts from *A. tsao-ko* by using free radical scavenging assays *in vitro* and D-glucose-induced mice intervention experiments *in vivo*. There were dose-effect relationships observed in the hypoglycemic and antioxidant properties of methanol extracts from *A. tsao-ko*. The functional mechanism of PMEAT and its impact on the human body have not yet been fully researched. Additional preclinical and clinical studies are required for the potential use of this natural herbal resource.

## Data Availability Statement

The original contributions presented in the study are included in the article/Supplementary Material, further inquiries can be directed to the corresponding author/s.

## Ethics Statement

The animal study was reviewed and approved by Ethics Committee of Shijiazhuang University (approve number is SJZXY 20200501).

## Author Contributions

LY designed and supervised the study. LX, DY, YL, HJ, JC, and LH were involved in animal experimentation. YL and HJ performed the statistical analyses and interpreted the data. LX and DY drafted the manuscript. JC revised the article critically for important intellectual content. All authors approved the final version of the manuscript to be submitted. All authors contributed to the article and approved the submitted version.

## Funding

This study was funded by the Foundation of the Department of Education of Hebei Province (QN2020525) and the Medical Science Research Project, Health Commission of Hebei Province (Key Science and Technology Research Program, No. 20210299).

## Conflict of Interest

The authors declare that the research was conducted in the absence of any commercial or financial relationships that could be construed as a potential conflict of interest.

## Publisher's Note

All claims expressed in this article are solely those of the authors and do not necessarily represent those of their affiliated organizations, or those of the publisher, the editors and the reviewers. Any product that may be evaluated in this article, or claim that may be made by its manufacturer, is not guaranteed or endorsed by the publisher.
