# Supplementary material for: Characterization, Hypoglycemic Activity, and Antioxidant Activity of Methanol Extracts From Amomum tsao-ko: in vitro and in vivo Studies
Source: Front Nutr. 2022 Jul 12;9:869749. doi: 10.3389/fnut.2022.869749 (PMC9315379; doi:10.3389/fnut.2022.869749)

Supplementary Figure 1. Study flow chart and design of in vivo hypoglycemic activity evaluation

Con, control group; HFD, high fat diet; Mod, model group; PMEAT-L, low dose PMEAT group; PMEAT-H, high dose PMEAT group.

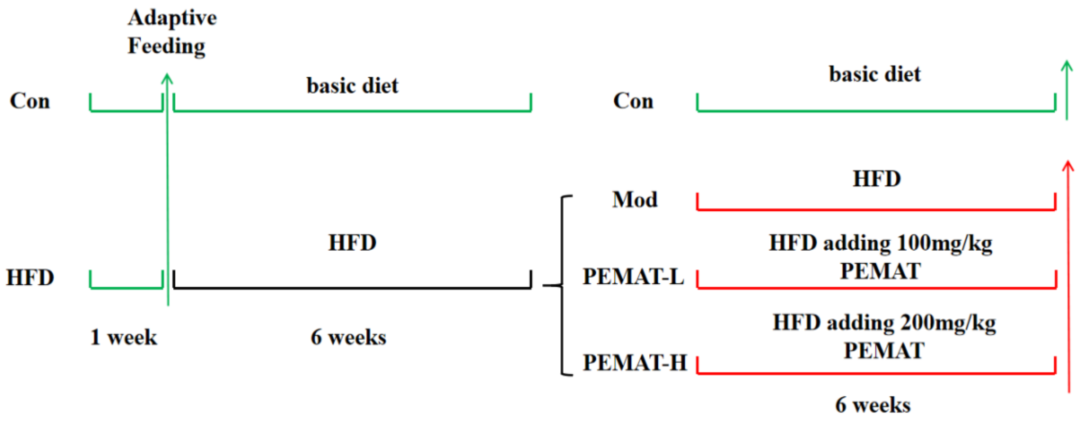

Supplementary Figure 2. Study flow chart and design of in vivo anti-oxidant activity evaluation

Con, control group; HFD, high fat diet; Mod, model group; PMEAT-L, low dose PMEAT group; PMEAT-H, high dose PMEAT group.

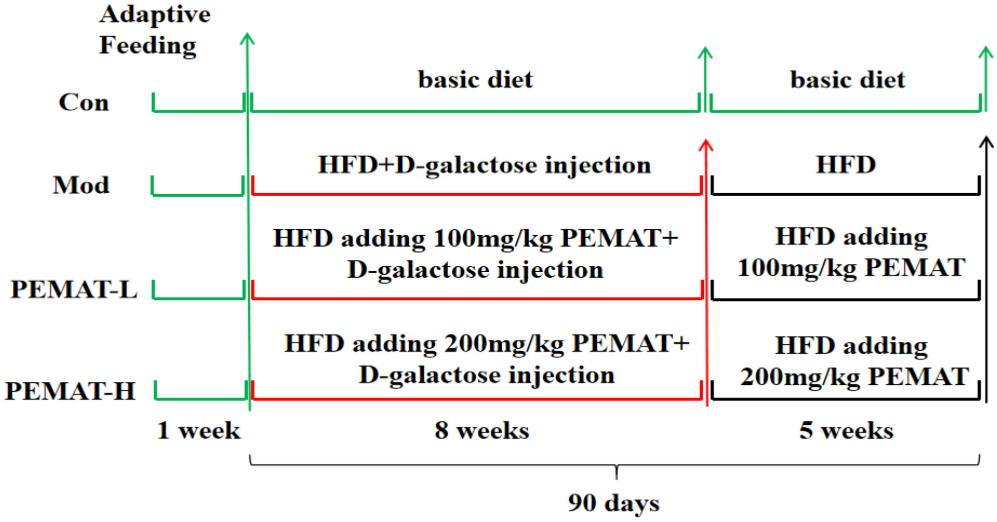

Supplement: Supplementary file 1 [file Presentation_1.pdf]
